# ADS024, a single-strain live biotherapeutic product of *Bacillus velezensis* alleviates dextran sulfate-mediated colitis in mice, protects human colonic epithelial cells against apoptosis, and maintains epithelial barrier function

**DOI:** 10.3389/fmicb.2023.1284083

**Published:** 2024-01-10

**Authors:** Sophie Irwin, Andrea Chupina Estrada, Becca Nelson, Ashlen Bullock, Berkeley Limketkai, Wendy Ho, Susan Acton, Laurent Chesnel, Hon Wai Koon

**Affiliations:** ^1^Vatche and Tamar Manoukian Division of Digestive Diseases, David Geffen School of Medicine at the University of California, Los Angeles, Los Angeles, CA, United States; ^2^Adiso Therapeutics Inc., Concord, MA, United States

**Keywords:** inflammation, anti-inflammatory, inflammatory bowel disease (IBD), GI, biologic, therapy, single-strain live biotherapeutic product, apoptosis

## Abstract

Epithelial cell apoptosis and compromised gut barrier function are features of inflammatory bowel disease. ADS024 is a single-strain live biotherapeutic product (LBP) of *Bacillus velezensis* under development for treating ulcerative colitis (UC). The cytoprotective effects of the sterile filtrate of ADS024’s secreted products on UC patient-derived colonic tissues, human primary colonic epithelial cells (HPEC), and human colonic epithelial T84 cells were evaluated. ADS024 filtrate significantly inhibited apoptosis and inflammation with reduced Bcl-2 Associated X-protein (BAX) and tumor necrosis factor (TNF) mRNA expression in fresh colonic explants from UC patients. Exposure to UC patient-derived serum exosomes (UCSE) induced apoptosis with increased cleaved caspase 3 protein expression in HPECs. ADS024 filtrate diminished the UCSE-mediated apoptosis by inhibiting cleaved caspase 3. TNFα and interferon-gamma (IFNγ) damaged epithelial barrier integrity with reduced transepithelial electrical resistance (TEER). ADS024 filtrate partially attenuated the TEER reduction and restored tight junction protein 1 (TJP1) expression. Oral live ADS024 treatment reduced weight loss, disease activity, colonic mucosal injury, and colonic expression of interleukin 6 (IL-6) and TNFα in dextran sodium sulfate (DSS)-treated mice with colitis. Thus, ADS024 may protect the colonic epithelial barrier in UC via anti-inflammatory, anti-apoptotic, and tight-junction protection mechanisms.

## Introduction

Inflammatory bowel disease (IBD), a chronic immune-related inflammation of the gastrointestinal tract, is generally categorized into Crohn’s disease (CD) and ulcerative colitis (UC). About 1 in 100 Americans are diagnosed with IBD (Centers for Disease Control and Prevention). The pathogenesis of UC and CD is believed to be an aberrant immune response, which may be mediated by genetics, gut microbiota, host immunology, and environmental factors (Guan, 2019). IBD patients can have different periods of symptomatic flares, remission, and complications, affecting their quality of life.

IBD is a lifelong disease without a complete cure. The treatments for IBD can include medication, changes in diet and nutrition, and surgery. Conventional IBD medications, including aminosalicylates, corticosteroids, immunomodulators, and biologics, facilitate and maintain remission (Cai et al., 2021). Some patients are irresponsive to the treatments, and the disease progresses. Therefore, new therapeutic strategies are being developed to improve the remission rate (Al-Bawardy et al., 2021).

As IBD is associated with gut dysbiosis, microbiota-based therapies have been explored in recent years. Fecal microbiota transplantation (FMT) can cure *C. difficile* infection with a 90% success rate, a frequent complication of IBD (Quraishi et al., 2017). However, the responses of IBD patients to FMT are highly variable (Allegretti, 2021). Some FMTs cause adverse effects (AE) in patients, creating safety concerns (Park and Seo, 2021). Alternatively, capsule-based standardized microbiota approaches are being developed as live biotherapeutic products (LBPs). Probiotics are also considered for managing IBD (Selvamani et al., 2022). As of today, no bacterial product has been approved for treating IBD.

In 2021, a new single-strain live biotherapeutic product (SS-LBP) called *Christensenella minuta* completed a phase I clinical trial with good efficacy for treating obesity and associated metabolic diseases (YSOPIA Bioscience, unpublished data). Another published report showed that the same bacterial strain could ameliorate colitis in mice by protecting intestinal epithelial barrier function (Relizani et al., 2022). Therefore, SS-LBPs may be explored for IBD therapeutic development.

Similarly, ADS024 is an SS-LBP of *Bacillus velezensis* under development. ADS024 was identified using a culture-based screen of aerobic spore-formers that resulted in the detection of *B. velezensis* isolates, which displayed zones of inhibition against *C. difficile* and *C. difficile* toxin degradation activity (O'Donnell et al., 2022). ADS024 also prevented toxin B-mediated epithelial injury in fresh human colonic explants and inhibited toxin B-mediated apoptosis in human colonic epithelial cells by inhibiting caspase 3 cleavage (Xie et al., 2022a). Oral treatment of ADS024 ameliorated colitis and prevented antibiotic-associated recurrence in *C. difficile*-infected mice (Murphy et al., 2023). ADS024 does not colonize in the colon and does not affect gut microbiota in miniature pigs (Murphy et al., 2023). ADS024 also lacks antimicrobial resistance genes and is susceptible to 18 antibiotics (Murphy et al., 2023). With its protective effects and good safety profiles, ADS024 is a good SS-LBP candidate to be evaluated as a potential treatment for IBD.

The cytoprotective effects of ADS024 against *C. difficile* infection inspired us to explore the therapeutic potential of ADS024 in IBD. We hypothesize that ADS024 can reduce apoptosis and protect tight junctions in colonic epithelial cells under conditions associated with UC, leading to disease amelioration. This study comprehensively evaluated the effect of ADS024 on UC patient-derived colonic explants, clinically relevant UC-related *in vitro* models, and a dextran sodium sulfate (DSS)-induced colitis murine model.

## Materials and methods

### ADS024 bacterial preparations

Bacteria were lyophilized in the presence of a cryoprotectant to stabilize ADS024. Lyophilized ADS024 material (lot #210421) was used in all *in vitro* experiments. Lyophilized ADS024 material (lot #230920) was used in the animal experiment. A cryoprotectant-containing control filtrate without bacteria (lot #180122) was used as the negative control. The bacterial stock was dissolved to 6×10^9^ colony forming units (cfu)/mL in RPMI1640 (pH 8) and incubated at 37°C for 30 min before use (Xie et al., 2022a). The bacterial suspensions were filtered with 0.22 μm filters to produce sterile filtrates (FS), which were further serially diluted to 1X, 0.1X, 0.01X, and 0.0001X in PBS (pH 7.4). 1 μL of the sterile filtrates were added to cells and tissue cultures (1 mL) to final titers equivalents of 6 × 10^6^, 6 × 10^5^, 6 × 10^4^, and 6 × 10^3^ cfu/mL. The bacterial filtrates were used within 1 h of resuspension to ensure freshness. Sterile filtration of ADS024 keeps the metabolites, proteins, or extracellular vesicles secreted by the live bacteria during the 30-min rehydration period while avoiding any solvent that might denature secreted membrane vesicles or proteins. The sterile filtration also prevented bacterial contamination of the cell culture incubator.

For molecular weight cut-off (MWCO) testing, the ADS024 filtrate was filtered by Vivaspin 2 MWCO columns (100 kDa: 45-001-570; 50 kDa: 45-001-569; 30 kDa: 45-001-568; 10 kDa: 45-001-567; 5 kDa:45-001-566; 3 kDa:45-001-565) from Cytiva (Xie et al., 2022a).

### Human serum samples

Serum samples from UC patients were prospectively collected from 2012 to 2015 (Tran et al., 2017). UCLA Institutional Review Board (IRB 12-001499) approved this study. UCLA Pathology obtained written informed consent from all subjects, so UCLA IRB waived a separate informed consent.

Sera were pooled from 6 UC patients to prepare serum exosomes (UCSE) by total exosome isolation reagent (#4478360, ThermoFisher; Wang et al., 2020). The protein concentrations in the UCSE were determined by bicinchoninic acid (BCA) protein assay (#23225, ThermoFisher).

### Fresh human colonic explants from UC patients

Fresh colonic tissues from UC patients with moderately inflamed histology from UCLA Surgical Pathology were obtained between 2021 and 2022. UCLA IRB (12-001499) approved the study. The fresh human colonic explants in 3 × 3 mm per piece were incubated in serum-free RPMI1640 media with or without 100 μg/mL UCSE for 30 min. Control and ADS024 filtrates were subsequently added and incubated for 24 h.

The baseline characteristics of intestinal tissues and serum samples are shown in Table 1.

Inclusion criteria: UC was diagnosed by UCLA’s gastroenterologists.
Exclusion criteria: Pregnant women, prisoners, minors under age 18, concurrent acute infection (cytomegalovirus infection, *C. difficile* infection, and tuberculosis), and malignant conditions were excluded.

**Table 1:** Baseline characteristics of UC patient-derived colonic explants, CD patient-derived ileal explants, UC patients for UCSE preparation, and UC patient-derived PBMCs.

**Fresh colonic tissues from UC patients**		
Patient	1	2
Age	50	40
Gender	Male	Female
Disease	UC	UC
Disease location	Transverse colon	Transverse colon
**Fresh ileal tissues from CD patients**		
Patient	1	2
Age	41	30
Gender	Male	Female
Disease	CD	CD
Disease location	ileum	ileum
**Serum samples for making UCSE (mean)**		
Age at collection		40
Gender (% male)		35
Partial Mayo Score		2.1
Duration of disease (years)		6
*n*		6
	**Stemcell Technologies UC PBMC 70051**	**UCLA UC PBMC**
Lot	RG1147	BK08
Age	43	30
Sex	Female	Male
Ethnicity	Caucasian	Caucasian
Diagnosis date	31-12-1994	08-04-2023
Process date	07-11-2019	10-04-2023
Smoker	no	no
Medications	Ambien, Khlonopin, Synthroid	mesalamine

### Human primary colonic epithelial cells

Human primary colonic epithelial cells (HPEC; H6047, Cell Biologics) were cultured in complete human epithelial medium (H6621, Cell Biologics) containing 10% fetal bovine serum and 1% penicillin–streptomycin to 80% confluence and then switched to serum-free media overnight (Xie et al., 2022a,b). Cells were subsequently pretreated with 1 μL/mL control and ADS024 sterile filtrates for 30 min, followed by 100 μg/mL UCSE and further incubated at 37°C for 24 h.

To quantify apoptosis, serum-starved HPECs in white-wall clear-bottom 96-well plates (165306, ThermoFisher) were treated with 100 μg/mL UCSE, followed by control sterile filtrate and ADS024 sterile filtrates at various dilutions and MWCOs. After adding UCSE and sterile filtrates, the cells were added with Promega RealTime-Glo Annexin V apoptosis assay reagents (JA1011, Promega) in a 1:1,000 ratio. After 24 h, the luminescence (apoptosis) signal was measured with a BioTek Synergy H1 plate reader (Xie et al., 2022a).

To identify apoptosis-related proteins, the serum-starved HPECs in 6-well plates were pretreated with sterile filtrates for 30 min, followed by 100 μg/mL UCSE for 24 h. The cell lysates (300 μg protein/group) were collected for Proteome Profiler Human Apoptosis Array Kit (ARY009, R&D Systems) and Human Apoptosis Signaling Array C1 (AAH-APOSIG-1-4, RayBiotech). All needed reagents were included in the kits. The signals were captured and analyzed using a Bio-Rad ChemiDoc Imaging system and Bio-Rad Image Lab software (Xie et al., 2022a).

### UC patient-derived peripheral blood mononuclear cells

A fresh blood sample from a UC patient were obtained through UCLA Pathology and isolated PBMCs with SepMate-15 (#85415, Stemcell Technologies). A vial of UC-PBMCs (#70051) was purchased from Stemcell Technologies. The baseline characteristics of UC-PBMC are shown in Table 1. The UC-PBMC was cultured in RPMI1640 medium with 10% fetal bovine serum and 1% penicillin–streptomycin. Secreted human interleukin 8 (IL-8) was detected by DuoSet ELISA kit (DY208) from R&D Systems.

### Epithelial barrier function measurement

The human colonic epithelial T84 cell monolayer was cultured on Transwell inserts (#3470, Corning) at 2 × 10^5^ cells/insert density and transepithelial electrical resistance (TEER) was measured with a dual-electrode connected to an epithelial volt/ohm meter (World Precision Instruments). When the measured TEER reached 2,000 Ω, 10 ng/mL tumor necrosis factor-alpha (TNFα) and 10 ng/mL interferon-gamma (IFNγ) were added to the basolateral side and ADS024 sterile filtrate to both sides. TEER was measured at 0 and 4–6 h. The average value was recorded as the measured TEER using the calculation: TEER (Ω·cm^2^) = (measured TEER–blank control TEER) × growth area (Xie et al., 2023).

For tight junction protein measurement, the serum-starved T84 cells were incubated with 10 ng/mL TNFα, 10 ng/mL IFNγ, and sterile filtrates for 24 h. Then, the cells were lyzed with radioimmunoprecipitation assay (RIPA) buffer (#89900, ThermoFisher) containing 1X protease inhibitor cocktail (#78429, ThermoFisher). Tight junction protein 1/TJP1 (MBS2605490), occludin (MBS2704294), and claudin 1 (MBS2704401) in the lysates were detected by ELISA kits from MyBioSource. Protein concentrations in cell lysates were determined by a bicinchoninic acid (BCA) assay (#23225, ThermoFisher).

### Animal study

Six to eight-week-old male C57BL/6 mice (Charles River Laboratories) with an average starting body weight (± standard deviation) of 21.0 ± 1.76 g were used. The animal rooms had HEPA air filtration at a temperature of 70 ± 5°F, 50% ± 20% relative humidity, 12–15 air changes per hour, and 12/12-h light/dark cycle. The facility staff swept and mopped the floor daily with a commercial detergent. They disinfected walls and cage racks with diluted bleach solution monthly. All workers always disinfected all surfaces and materials introduced into the hood with a commercial disinfectant.

Mice were acclimatized for 3 days and then randomized into four groups at the start of the study: one group of six mice, one group of 15 mice, and two groups of 12 mice each. Each mouse was identified by an ear punch corresponding to an individual number. The mice were housed in cages with sterile Bed-o’Cobs® or equivalent bedding, Labdiet 5,053 sterile rodent diet, and reverse osmosis-purified water *ad libitum*. The facility washed cages, tops, and water bottles with a commercial detergent, followed by air drying. Each cage card shows information to identify the study, dose, animal number, and treatment group.

Colitis in mice was induced by 3% dextran sulfate (DSS; MP Biomedicals, Cat #0260110) in drinking water from day 0 to day 5. Control naïve mice did not receive DSS. Mice were dosed by oral gavage of 5 × 10^8^ CFU ADS024 in phosphate-buffered saline (PBS) twice daily starting on Day 6 for 14 days. Negative control mice were dosed with PBS (vehicle). All interventions were performed during the light cycle.

Mice were monitored daily for weight loss, diarrhea, and blood in the stool. On day 19, all mice were sacrificed for histology assessment of the colon. Colonic cytokines and myeloperoxidase (MPO) levels were measured by ELISA. Distal colon samples from 44 mice (88 pieces), fixed in 10% neutral buffered formalin, were shipped to Inotiv Boulder (formerly HistoTox Labs) for hematoxylin and eosin (H&E) staining and evaluation by a board-certified veterinary pathologist using light microscopy. Each sample was trimmed into three transverse sections and embedded together in a single block (six pieces of tissue each with proximal toward the label and distal away from the label). Three slides were sectioned from each block at 5 μm and stained with hematoxylin and eosin (H&E). Lesions were scored according to severity 0–5 (0 = not present/normal, 1 = minimal, 2 = mild, 3 = moderate, 4 = marked, 5 = severe). Scored features were added together for each sample to obtain a sum colitis score (Range: 0–25). For cytokine and MPO measurements, a piece of the colon from each animal was rinsed and snap-frozen. The colon tissue was homogenized in PBS plus protease inhibitor. The resulting homogenate was centrifuged to obtain a clear supernatant containing the total tissue protein. This colon tissue supernatant was used to determine the concentrations of interleukin 1beta (IL-1β), interleukin 6 (IL-6), interleukin 10 (IL-10), and tumor necrosis factor-alpha (TNF-ɑ) via multiplex (Luminex). Colon MPO concentrations were also determined in the same groups by ELISA. The mouse experiment was conducted at Biomodels, Waltham, MA, USA.

### Statistical analysis

All experiments were repeated to ensure reproducibility. Two data groups were compared with unpaired Student’s t-tests and multiple data groups with ordinary one-way ANOVAs using GraphPad Prism. Results were expressed as mean +/− standard deviation (SD) or standard error of means (SEM). Significant *p* values in each figure are indicated.

### Data availability statement

Additional unpublished data from the study may be shared. Please contact HK or Adiso Therapeutics (https://adisotx.com/).

## Results

### ADS024 filtrate prevented apoptotic and inflammatory gene expression in UC patient-derived fresh colonic explants

**Figure 1:**
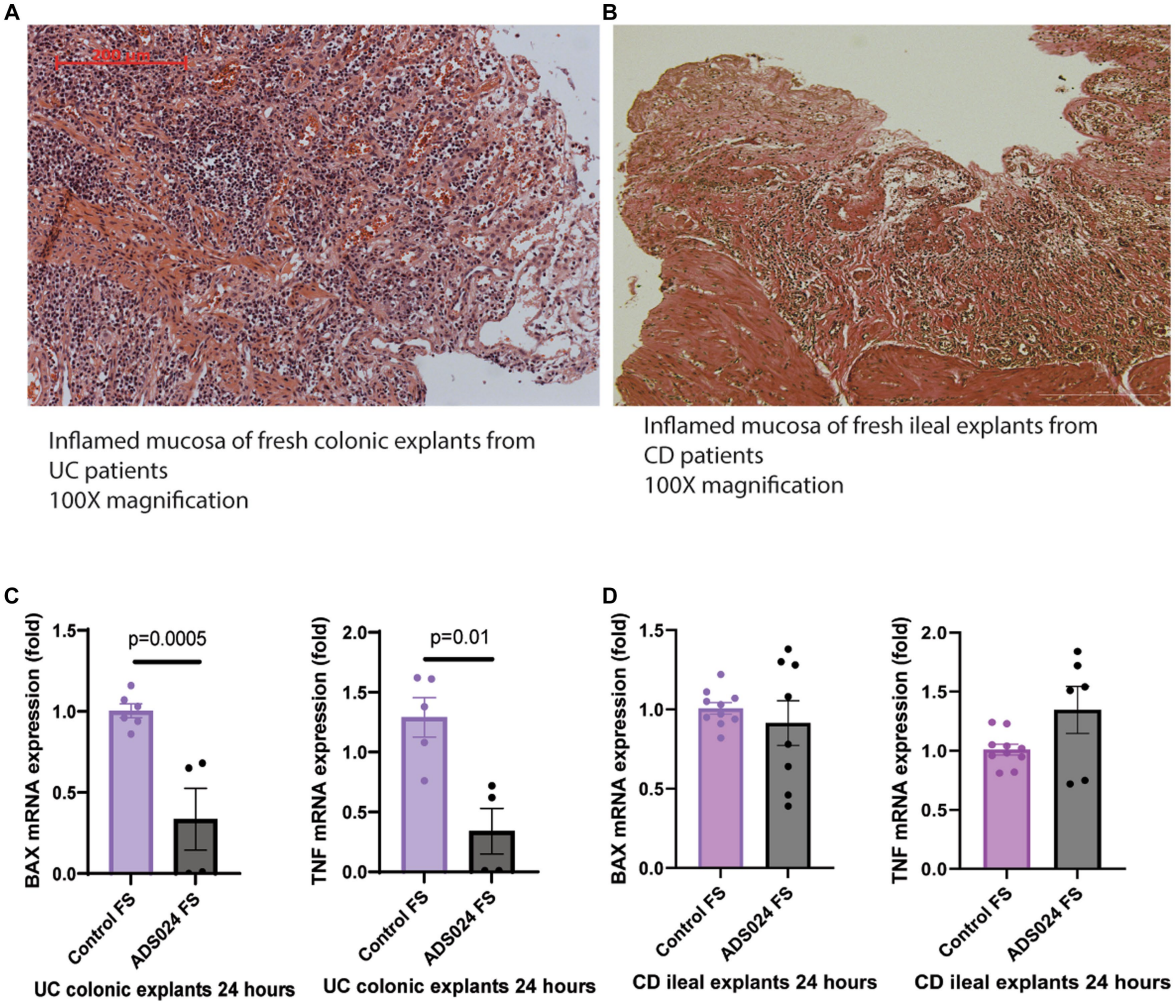
ADS024 exerted direct anti-apoptotic and anti-inflammatory effects in UC patient-derived fresh colonic explants. **(A)** H&E-stained image of a representative fresh colonic explant from 2 UC patients. A red 200-micron bar is in the top left corner. **(B)** H&E-stained image of a representative fresh ileal explant from 2 CD patients. A white 200-micron bar is in the lower right corner. Some differences in stained color are normal and do not affect evaluation. Both specimens showed epithelial injury with numerous infiltrating immune cells. **(C)** BAX and TNF mRNA expression in the UC colonic explants after 24-h incubation with control sterile filtrate and ADS024 sterile filtrate at 1X. Results were pooled from 2 UC patients. **(D)** BAX and TNF mRNA expression in the CD ileal explants after 24-h incubation with control sterile filtrate and ADS024 sterile filtrate at 1X. Results were pooled from 2 CD patients. Each patient’s explant was cut into multiple pieces if possible. Each sample was run in duplicate in the real-time RT-PCR. Mean ± SD. Student *t*-tests were used.

Our previous report demonstrated that ADS024 sterile filtrate prevented mucosal injury in *C. difficile* toxin B-exposed fresh human colonic explants (Xie et al., 2022a). Here, this study explored the direct protective effects of ADS024 sterile filtrate in fresh colonic explants from UC patients. The colonic mucosa of UC patients and the ileal mucosa of CD patients are characteristic of disrupted mucosal structures with numerous infiltrating immune cells (Figures 1A,B).

Compared to the control group, ADS024 filtrate treatment significantly reduced mRNA expression of pro-apoptotic Bcl-2 Associated X-protein (BAX) and pro-inflammatory tumor necrosis factor (TNF) in the fresh colonic explants from UC patients (Figure 1C), suggesting potential protective effects in UC. The 24-h treatment with ADS024 filtrate could not reverse the preexisting histological mucosal injury in the UC colonic explants (data not shown). ADS024 filtrate treatment did not impact the mRNA expression of BAX and TNF in the fresh CD patient-derived ileal explants (Figure 1D), suggesting a UC-specific protective effect.

### ADS024 filtrate prevented UCSE-mediated apoptosis in colonic epithelial cells

**Figure 2:**
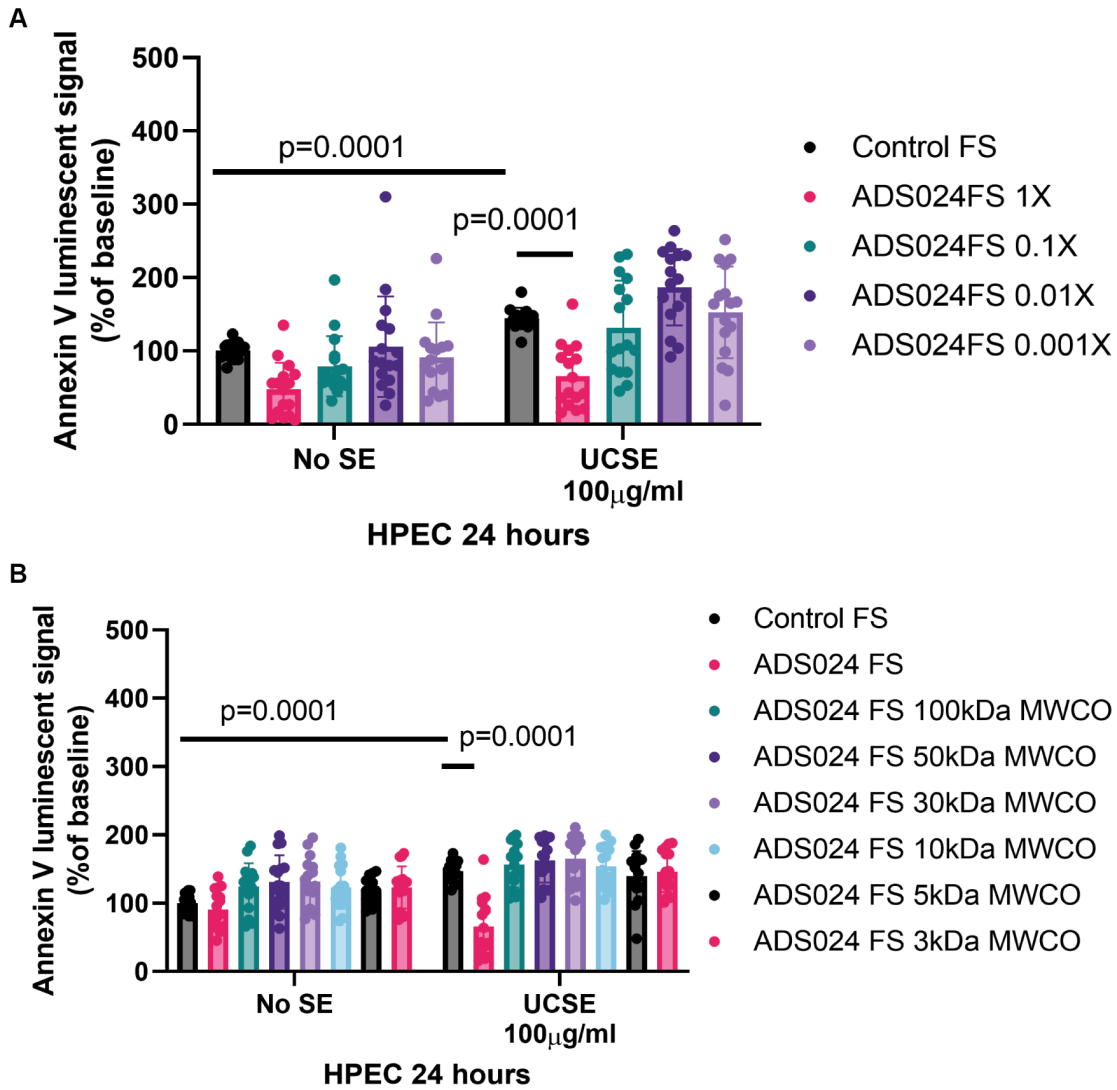
ADS024 sterile filtrate exerted anti-apoptotic effects in human colonic epithelial cells. **(A,B)** Apoptosis assays. The Annexin V luminescent signal above 100% indicated apoptosis. **(A)** UCSE treatment increased apoptosis in HPECs, which was reduced by ADS024 FS 1X. **(B)** UCSE treatment increased apoptosis in HPECs, which was only reduced by ADS024 sterile filtrate 1X without MWCO. The results were pooled from 3 to 4 independent experiments. Mean ± SD. One-way ANOVA tests were used.

Exposure to UCSE induced apoptosis in HPECs (Figure 2A), simulating a UC environment *in vitro*. Compared to the control, the ADS024 filtrate at 1X prevented UCSE-mediated apoptosis in HPEC. Further dilutions of ADS024 lost their anti-apoptotic effect (Figure 2A).

The molecular weight of the potential anti-apoptotic agent(s) in the ADS024 filtrate was estimated by filtering the ADS024 filtrates with MWCO columns. ADS024 filtrates at MWCO at 100 kDa and below did not exert anti-apoptotic effects in the UCSE-treated HPECs (Figure 2B). Therefore, the anti-apoptotic agent(s) in ADS024 is likely to be at least 100 kDa.

### ADS024 prevented UCSE-mediated apoptosis via inhibition of caspase 3 cleavage

**Figure 3:**
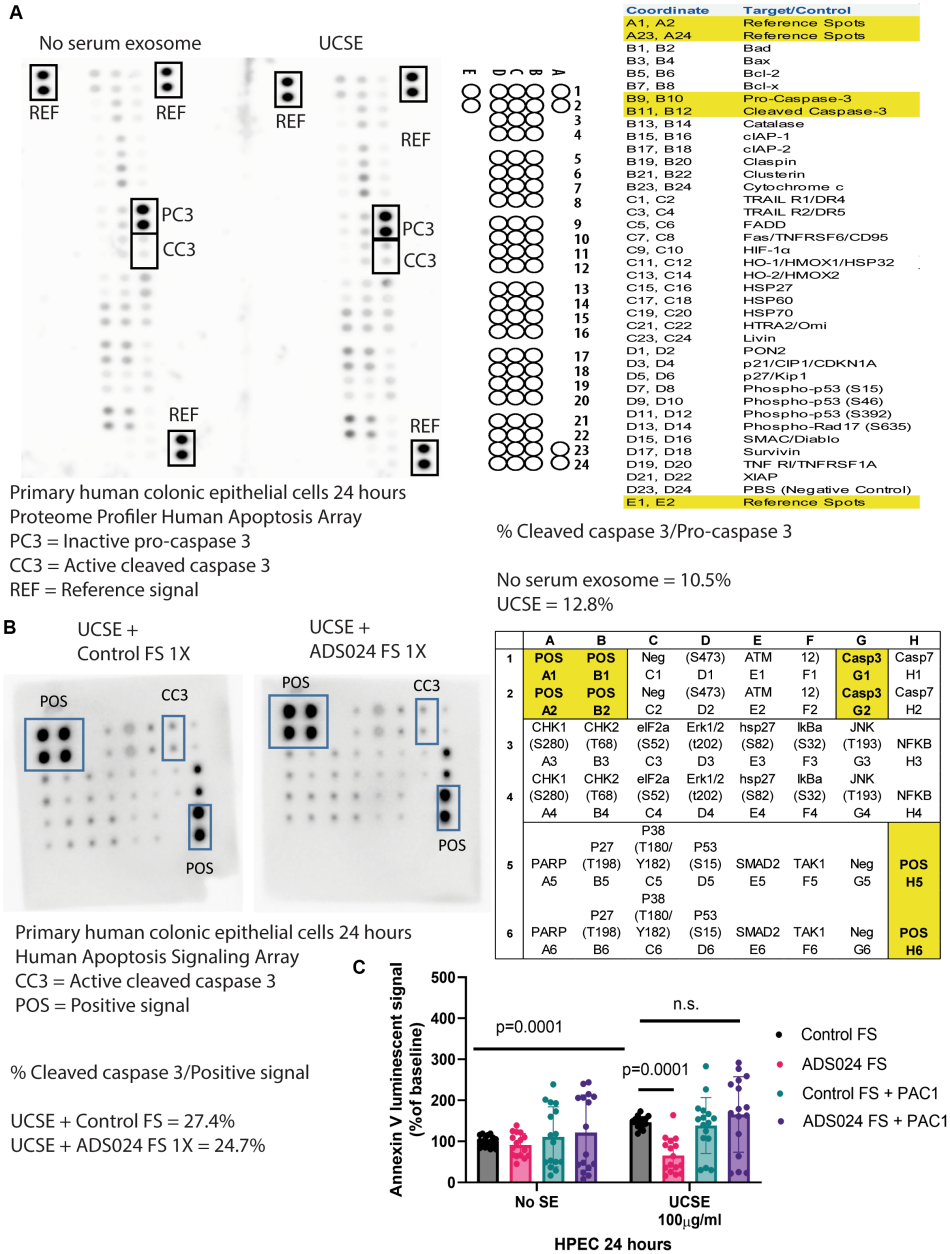
ADS024 inhibited apoptosis by inhibiting cleaved caspase 3 expression in human colonic epithelial cells. **(A)** Human apoptosis array. Serum-starved HPECs were treated with 100 μg/mL UCSE for 24 h. The cells were collected for the Proteome Profiler Human Apoptosis Array (ARY009, R&D Systems). **(B)** Human apoptosis signaling array. Serum-starved HPECs were pretreated with ADS024 sterile filtrate at 1X dilution for 30 min, followed by 100 μg/mL UCSE for 24 h. The cells were collected for the Proteome Profiler Human Apoptosis Array (AAH-APOSIG-1-4, RayBiotech). **(C)** Apoptosis assays. Serum-starved HPECs were pretreated with sterile filtrate of ADS024 at 1X dilution with or without adding 10 μM procaspase activating compound 1/PAC1 (#10009317, Cayman Chemical). PAC1 is an activator of caspase 3 cleavage. Promega RealTime-Glo Annexin V apoptosis assay reagents in a 1:1000 ratio were added simultaneously. Thirty minutes later, 100 μg/mL UCSE was added to induce the apoptosis process for 24 h. PAC1 reversed the ADS024 filtrate-mediated inhibition of UCSE-dependent apoptosis. Results were pooled from 4 independent experiments. Mean ± SD. One-way ANOVA tests were used.

A protein array was utilized to detect 35 anti-/pro-apoptotic proteins in UCSE-treated HPECs quantitatively. UCSE increased activated pro-apoptotic cleaved caspase 3 protein expression level (Figure 3A). Another protein array was utilized to detect 19 apoptosis-signaling proteins in UCSE-treated HPECs. Consistent with our previous *C. difficile* toxin B study (Xie et al., 2022a), ADS024 sterile filtrate at 1X reduced the protein level of cleaved caspase 3 in UCSE-treated HPECs (Figure 3B).

The anti-apoptotic effect of ADS024 sterile filtrate against UCSE was attenuated by a caspase 3 activator (PAC1; Figure 3C). This finding suggested that ADS024 filtrate exerts an anti-apoptotic effect against UCSE by inhibiting caspase 3 cleavage in human colonic epithelial cells.

### ADS024 prevented UCSE-mediated epithelial barrier function disruption by restoring TJP1 expression

**Figure 4:**
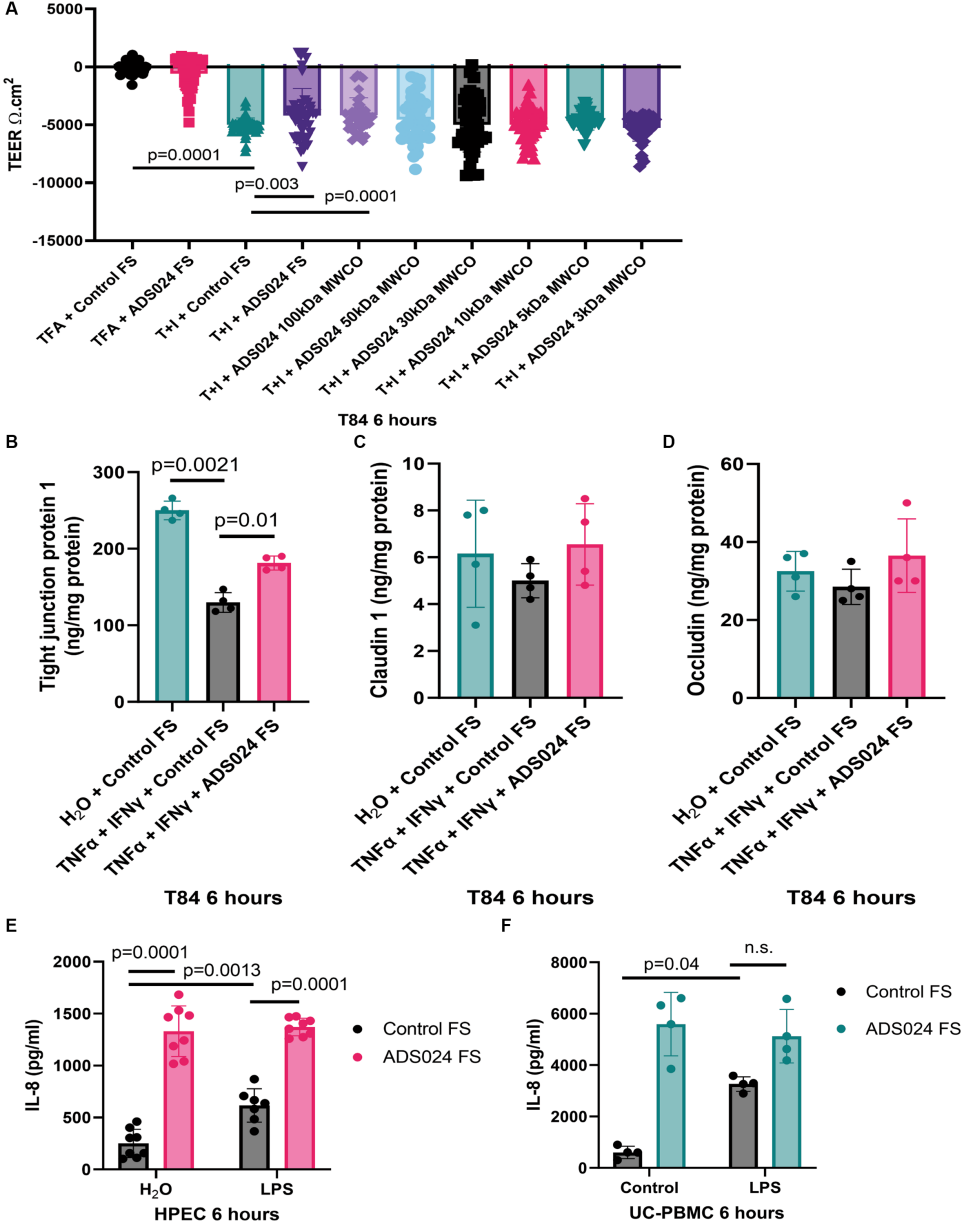
ADS024 protected epithelial barrier function in human colonic epithelial cells. **(A)** Epithelial barrier function test. T84 monolayers were grown on Transwell inserts. 10 ng/mL TNFα and 10 ng/mL IFNγ caused a reduction of TEER in 6 h, indicating loss of epithelial barrier function. The reduction was prevented by 1X ADS024 sterile filtrates pretreatment without MWCO and with 100 kDa MWCO. Results were pooled from 6 experiments. Each experiment had 6 readings. **(B–D)** Serum-starved T84 cells were pretreated with ADS024 sterile filtrate at 1X, followed by 10 ng/mL TNFα and 10 ng/mL IFNγ. Six hours later, TJP1, claudin 1, and occludin proteins in the lysates were determined by ELISA. Results were pooled from 4 independent experiments. Mean ± sd. One-way ANOVA tests were used. **(E,F)** IL-8 ELISA. HPECs and UC-PBMCs were pretreated with ADS024 sterile filtrate at 1X, followed by 10 ng/mL lipopolysaccharide. Six hours later, we determined IL-8 levels in the conditioned medium by ELISA. ADS024 sterile filtrate did not affect LPS-mediated IL-8 secretion in HPEC and UC-PBMCs. Results were pooled from 4 independent HPEC experiments and UC-PBMCs from 2 UC patients. Mean ± SD. One-way ANOVA tests were used. TFA, 0.1% trifluoroacetic acid (as a vehicle to dissolve TNFα and IFNγ). T + I, TNFα and IFNγ.

Disruption of epithelial barrier function leads to gut bacteria and proinflammatory substances penetrating the mucosal layer and triggering inflammation in UC. Consistent with another study (Fischer et al., 2013), TNFα and IFNγ caused epithelial barrier dysfunction with reduced transepithelial electrical resistance (TEER; Figure 4A). A lowered TEER indicated more electrical current flowing through the leaky epithelial layer.

Adding ADS024 filtrate did not affect baseline TEER (Figure 4A). However, ADS024 filtrate at 1X significantly diminished the reduction of TEER in TNFα- and IFNγ-treated T84 cells, indicating a partial restoration of epithelial barrier function (Figure 4A). The molecular weight of the potential cytoprotective agent(s) in the ADS024 filtrate was estimated by MWCO column filtration. Only filtrates of 100 kDa or above exhibited the cytoprotective effects (Figure 4A).

Three common tight junction-related proteins were quantified by ELISA. TNFα and IFNγ treatment significantly reduced TJP1 expression in T84 cells, and expression was partially restored by exposure to ADS024 sterile filtrate at 1X (Figure 4B). Neither TNFα and IFNγ nor ADS024 sterile filtrate affected claudin 1 and occludin protein expression in T84 cells (Figures 4C,D).

### ADS024 filtrate did not affect lipopolysaccharide-mediated IL-8 secretion

IL-8 is a chemokine to induce chemotaxis in immune cells (Bickel, 1993). UC patient-derived PBMCs (UC-PBMC) and HPECs secrete high levels of proinflammatory IL-8. Lipopolysaccharide (LPS), a bacterial cell wall component, stimulated IL-8 secretion in UC-PBMCs and HPEC (Figures 4E,F). ADS024 filtrate dramatically increased IL-8 secretion when alone and in the presence of LPS treatment (Figures 4E,F). Thus, ADS024 filtrate does not possess direct anti-IL-8 effects in immune and epithelial cells.

### Oral live ADS024 treatment ameliorated colitis in DSS-treated mice

**Figure 5:**
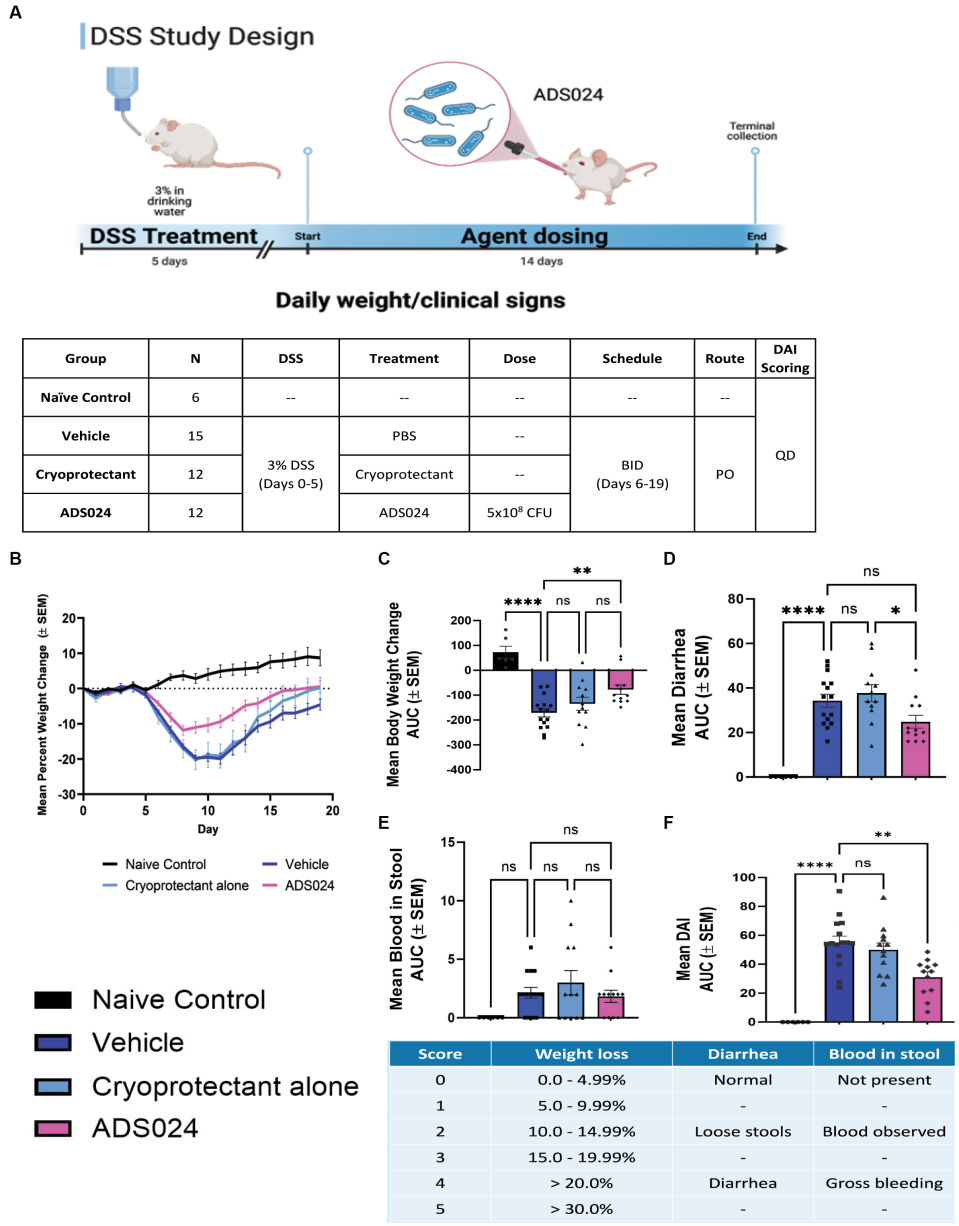
Oral live ADS024 treatment reduced weight loss and disease activity in mice with colitis. (**A**, upper) Experimental plan of mouse DSS colitis experiment. This figure was created with BioRender.com. (**A**, lower) Plan of mouse treatment and monitoring. **(B)** Mean percent body weight change. **(C)** Mean percent body weight change from day 0 to day 20 in area-under-curve (AUC) format. **** indicates *p* < 0.0001 vs. DSS. * indicates *p* < 0.05 vs. DSS. Ns indicates insignificant when compared to DSS. **(D)** Mean diarrhea change from day 0 to day 20 in AUC format. **** indicates *p* < 0.0001 vs. DSS. **(E)** Mean blood in stool from day 0 to day 20 in AUC format. **(F)** The mean of disease activity index (DAI) changes in AUC format. **** indicates *p* < 0.0001 vs. DSS. * indicates *p* < 0.01 vs. DSS. The scheme of disease activity index is shown below. *n* = 6 mice in naïve/control group; 15 mice in DSS; 12 mice in DSS + ADS024. Mean ± SEM. One-way ANOVA tests were used. BID = twice a day. PO = by mouth, orally.

The protective effects of ADS024 were further evaluated by inducing UC-like colitis in mice with DSS, followed by 14 days of oral live ADS024 treatment and monitoring until day 20 (Figure 5A). DSS colitis caused the most severe weight loss in mice, significantly minimized by live ADS024 treatment (Figure 5B). The colitis was comprehensively assessed with a disease activity index (DAI) that evaluates weight loss, diarrhea, and bloody stool (Figures 5C–F). DSS colitis caused weight loss, diarrhea, and bloody stool, resulting in a significant increase in DAI, which was reduced by oral live ADS024 treatment (Figures 5C–F). The cryoprotectant alone did not affect the DSS-mediated weight loss, diarrhea, bloody stool, and DAI (Figures 5C–F).

**Figure 6:**
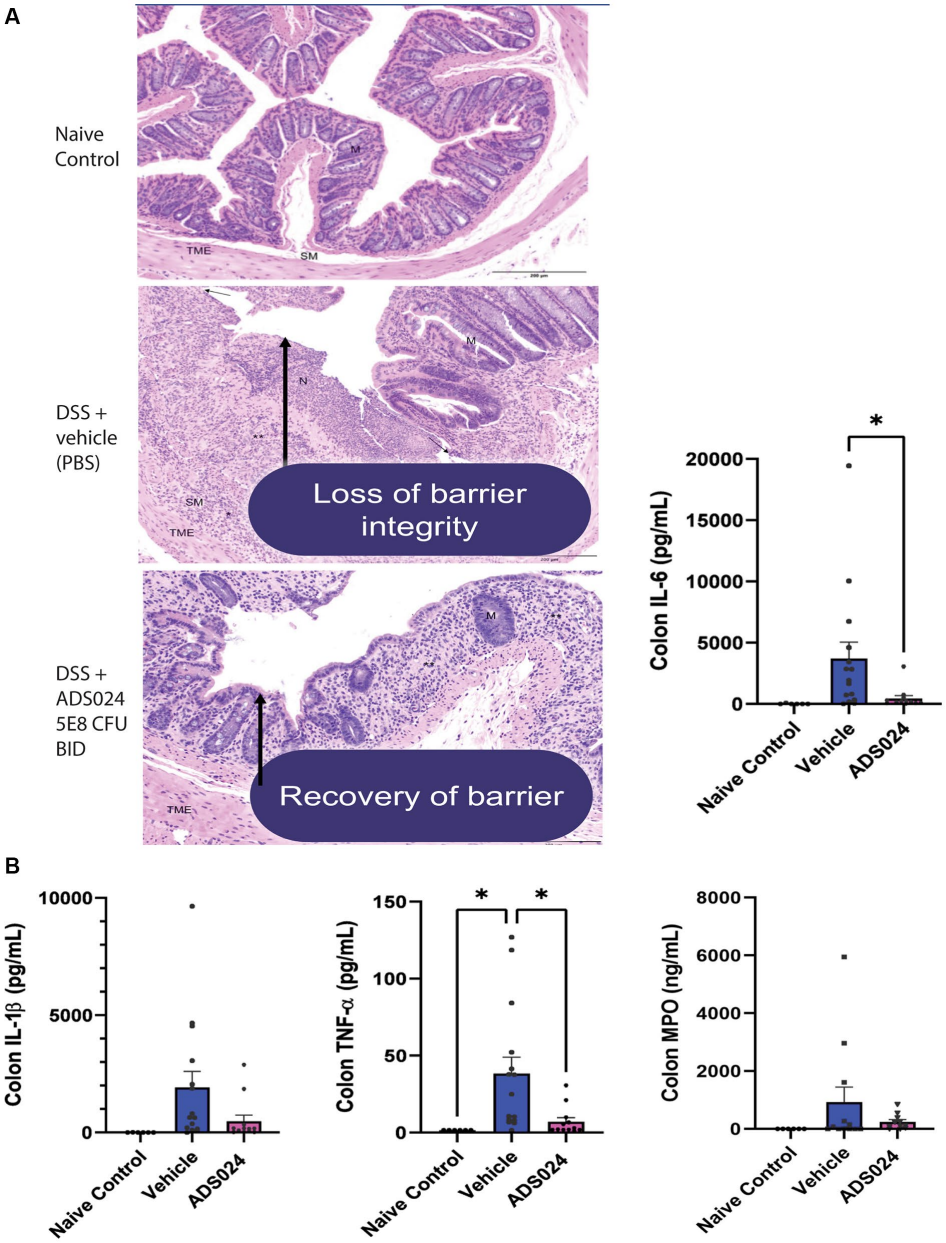
Oral live ADS024 treatment restored the colonic mucosal barrier and reduced colonic IL-6 expression in DSS-treated mice. **(A)** Representative H&E-stained images of mouse colonic mucosa on day 19. Black 200-micron scale bars are in the lower right corner. (High-resolution images with detailed descriptions are attached in Supplementary material.) DSS treatment caused mucosal disruption with immune cell infiltration, partially reduced by live ADS024 treatment. **(B)** Colonic IL-6, IL-1β, TNFα, and MPO protein levels were determined by ELISA. Live ADS024 significantly reduced colonic IL-6 levels in DSS-treated mice. ^*^p < 0.05, compared to the DSS vehicle group. *n* = 6 mice in naïve/control group; 15 mice in DSS; 12 mice in DSS + ADS024. Mean ± SEM. One-way ANOVA tests were used.

Compared to water-treated control, DSS treatment caused severe colitis exhibiting the expected histologic lesions of subacute inflammation, mucosal necrosis/gland loss, erosions, submucosal edema, and epithelial hyperplasia with infiltration of neutrophils, lymphocytes, plasma cells, and macrophages into the mucosa or submucosa. These observations are characteristic of the loss of colonic mucosal structure and immune cell infiltration, indicating the loss of barrier integrity (Figure 6A). Oral live ADS024 treatment partially improved mucosal structure and reduced infiltrating immune cells (Figure 6A).

DSS treatment increased colonic levels of several representative pro-inflammatory markers, including IL-6, IL-1β, TNFα, and myeloperoxidase (MPO) in mice (Figure 6B). Of note, oral live ADS024 treatment significantly reduced colonic IL-6 and TNFα, but not IL-1β, and MPO, levels in the DSS-treated mice (Figure 6B). Therefore, oral live ADS024 treatment reduced DSS-mediated weight loss, clinical disease activity, colonic mucosal injury, and select colonic pro-inflammatory cytokine expression in mice.

## Discussion

This is the first study to explore the direct protective effect of the SS-LBP ADS024 in UC using patient-derived colonic explants, human colonic epithelial cells, and DSS-treated animals. ADS024 filtrate inhibits apoptosis and protects epithelial barrier functions in colonic epithelial cells. Live ADS024 treatment significantly attenuates DSS-induced colitis severity.

ADS024 live bacteria secrete proteases, metabolites, and extracellular vesicles (Xie et al., 2022a). A molecular weight cut-off study showed that the anti-apoptotic agents were above 100 kDa (Figure 2B), while barrier-protective agents were above 50 kDa (Figure 4A). As ADS024 encodes multiple proteins, the collective effects of numerous protective proteins in ADS024 likely mediate its beneficial effects. In our previous study, ethyl acetate and isopropanol extraction processes inactivated proteases in the ADS024-secreted products (Xie et al., 2022a). Similarly, ethyl acetate and isopropanol extracts of ADS024 did not possess anti-apoptotic effects in UCSE-treated HPEC (data not shown). Therefore, the protective agents in ADS024 filtrate are likely to be proteins.

Exposure to high concentrations of ADS024 (1-0.01X) diminished cell viability in human colonic epithelial cells (Xie et al., 2022a). As ADS024 filtrate contains toxin B-degrading proteases, this inhibitory effect became insignificant when the ADS024 filtrate was diluted to 0.0001X (Xie et al., 2022a). However, colonic explants tolerated the proteases in 1X ADS024 filtrate (Xie et al., 2022a). Many probiotic strains, such as *Saccharomyces boulardii*, also produce proteases without causing detrimental effects on human colonic mucosa (Castagliuolo et al., 1999). Thus, ADS024-derived proteases do not significantly affect intestinal cell health.

Protease activities are increased in active UC colons (Galipeau et al., 2021). The diverse roles of proteases in UC are under active investigation. Some proteases cleave protease-activated receptor 2 (PAR2) and activate the inflammation pathway, exacerbating colitis (Kim et al., 2003). Although PAR2 antagonists showed protective effects in colitis (Lohman et al., 2012), no clinical trial demonstrated the therapeutic effect of broad-spectrum protease inhibitors among IBD patients. We are unable to conclude whether proteases mediated the protective effects of ADS024.

Apoptosis-mediating caspase 3 regulates DNA fragmentation and changes in cell morphology (Janicke et al., 1998). Although the mechanistic role of caspase 3 in IBD patients has not been fully elucidated, inhibition of colonic apoptosis is associated with reduction of colonic caspase 3 expression in mouse models of IBD colitis (Che et al., 2019; Magalhaes et al., 2023). On the other hand, activation of caspase 3 causes increased apoptosis, disruption of tight junction protein 1/ZO-1, and increased epithelial barrier permeability in colonic epithelial cells (Chin et al., 2006). It is possible that caspase 3 activation adversely affects gut barrier function in IBD.

Ethyl acetate extract of ADS024 could inhibit caspase 3 cleavage in toxin B-exposed HPEC (Xie et al., 2022a), suggesting metabolites may play a role. On the other hand, high molecular weight proteins in ADS024 filtrate exerted anti-apoptotic effects in UCSE-treated HPEC (Figure 2B). Thus, the ADS024-derived caspase 3-inhibiting agents against *C. difficile* toxin B and UCSE are likely different.

The pathogenic mechanisms of UC and CD are different (Ruiz Castro et al., 2021). ADS024 inhibited BAX and TNF mRNA expression in UC patient-derived colonic tissues but not CD patient-derived ileal tissues (Figure 1). ADS024 filtrate could also not reduce CD patient-derived serum exosome (CDSE)-mediated apoptosis in HPECs (data not shown). Therefore, the protective agents in the ADS024 secreted products affected the UC-specific signaling mechanism that maintains cell health and epithelial barrier function. As multiple proteins in the ADS024-secreted products may act synergistically, identifying the protective agents from ADS024 will require a significant and lengthy effort. Thus, the present manuscript cannot indicate the specific ADS024-derived proteins responsible for this UC-specific protection but has shown a minimum molecular weight.

For the clinical application of ADS024 in UC, it was shown that oral live ADS024 bacterial treatment successfully ameliorated DSS colitis in mice and with reduced weight loss, disease activity, mucosal injury, and colonic IL-6 and TNFα expression (Figures 5, 6). IL-6 is a proinflammatory cytokine correlated to clinical disease activity in IBD (Mavropoulou et al., 2020). An anti-IL-6 antibody (Olamkicept) recently successfully treated UC patients in a clinical trial (Zhang et al., 2023), suggesting IL-6’s involvement in UC pathogenesis. TNFα is also a target of UC as anti-TNF antibodies such as infliximab can modulate UC disease activity (Feuerstein et al., 2020). ADS024 may not possess direct anti-inflammatory effects as it did not affect pro-inflammatory chemokine IL-8 secretion in LPS-treated HPEC and UC patient-derived PBMC (Figures 4E,F). We speculate that ADS024 indirectly inhibits inflammation via epithelial barrier protection.

The mRNA expression of many tight junction proteins, including TJP1, was significantly reduced in the colons of UC patients (Kuo et al., 2021). TNFα and IFNγ caused a disruption of epithelial barrier function with reduced expression of the TJP1 gene or ZO-1 protein (Figure 4). Intestinal epithelial-specific ZO-1 deficient mice are healthy, suggesting that it is not essential to maintain normal gut barrier function (Kuo et al., 2021). However, these mice are hypersensitive to DSS colitis with defective mucosal repair and increased colonic caspase 3 cleavage and IL6 mRNA expression (Kuo et al., 2021). ADS024-regulated TJP1 protects mucosa and indirectly reduces apoptosis and immune activation in UC conditions. It is noted that ADS024 reduced TJP1/ZO-1 protein expression in T84 cells without TNFα and IFNγ treatment (Figure 4B), but it is not a concern because the same treatment did not affect TEER function (Figure 4A).

In summary, ADS024-secreted products (possibly proteins) inhibited UC-related apoptosis in human colonic tissues and epithelial cells by preventing caspase 3-dependent apoptosis. ADS024 secreted products also protect epithelial barrier function by maintaining tight junction protein 1. Live ADS024 bacterium ameliorated DSS colitis in mice with reduced colonic injury and inflammation. These findings support further investigations of ADS024 in UC patients.

## Data availability statement

The original contributions presented in the study are included in the article/supplementary material, further inquiries can be directed to the corresponding author.

## Ethics statement

The studies involving humans were approved by UCLA Institutional Review Board. The studies were conducted in accordance with the local legislation and institutional requirements. The participants provided their written informed consent to participate in this study.

## Author contributions

SI: Methodology, Writing – review & editing. AC: Methodology, Writing – review & editing. BN: Methodology, Writing – review & editing. AB: Methodology, Writing – review & editing. BL: Resources, Writing – review & editing. WH: Resources, Writing – review & editing. SA: Formal analysis, Funding acquisition, Methodology, Resources, Writing – original draft, Writing – review & editing. LC: Formal analysis, Funding acquisition, Methodology, Resources, Writing – original draft, Writing – review & editing. HK: Conceptualization, Funding acquisition, Investigation, Resources, Supervision, Validation, Writing – original draft, Writing – review & editing.
