# Supplementary material for: ADS024, a single-strain live biotherapeutic product of Bacillus velezensis alleviates dextran sulfate-mediated colitis in mice, protects human colonic epithelial cells against apoptosis, and maintains epithelial barrier function
Source: Front Microbiol. 2024 Jan 10;14:1284083. doi: 10.3389/fmicb.2023.1284083 (PMC10806143; doi:10.3389/fmicb.2023.1284083)

REPRESENTATIVE PHOTOMICROGRAPHS

Images are representative of group mean sum colitis scores and mean immunolabeling scores in the distal colon as among-group differences were better developed in this segment. Scale bars indicate 200 µm.

Image 1. (No DSS), Animal 1 Distal Colon. H&E, 100x. Non-lesioned colon is captured. The mucosa (M), artifactually separated submucosa (SM), and the tunica muscularis externa (TME) are indicated.

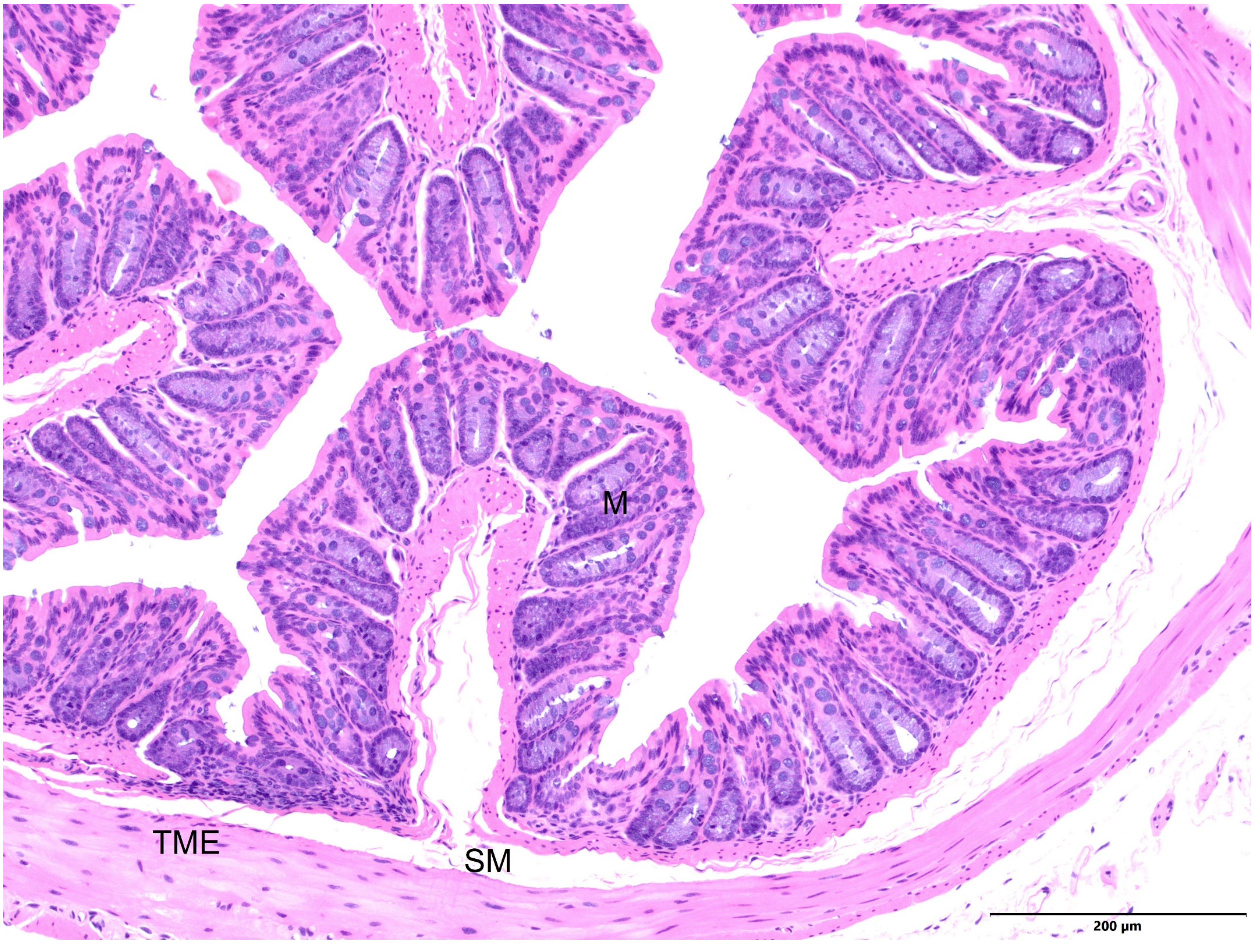

Image 2. (3% DSS + Vehicle), Animal 10 Distal Colon. H&E, 100x. A regionally extensive area of gland loss with infiltration of neutrophils, lymphocytes, and macrophages (**) is overlain by eroded surface epithelium (black arrows demarcate erosion), with luminal exudation of neutrophils (N). The adjacent mucosal glands (M) are hyperplastic, with gland elongation, crypt branching, reduced goblet cells, and increased mitotic figures observed. Inflammatory cells (*) also extend into the submucosa (SM). The tunica muscularis externa (TME) is indicated.

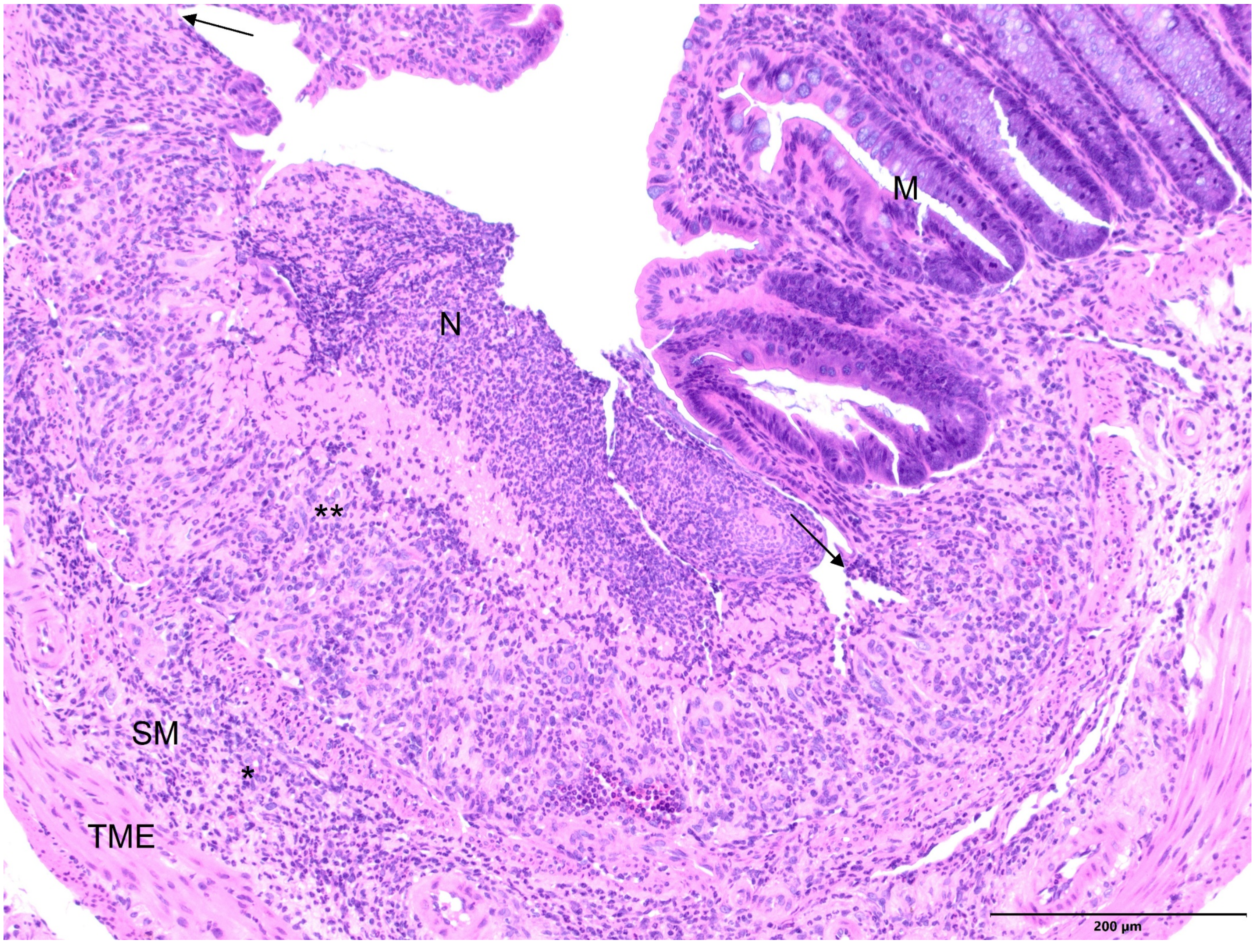

Image 3. (3% DSS + ART24 5x10^8/dose), Animal 52 Distal Colon. H&E, 100x. Similar to the other ART24-treated sample, areas of inflammatory cell infiltration and gland necrosis/loss (**) are overlain by intact surface epithelium. Modest hyperplasia is observed in the mucosal glands (M) adjacent areas of loss. The submucosa (SM) and the tunica muscularis externa (TME) are indicated.

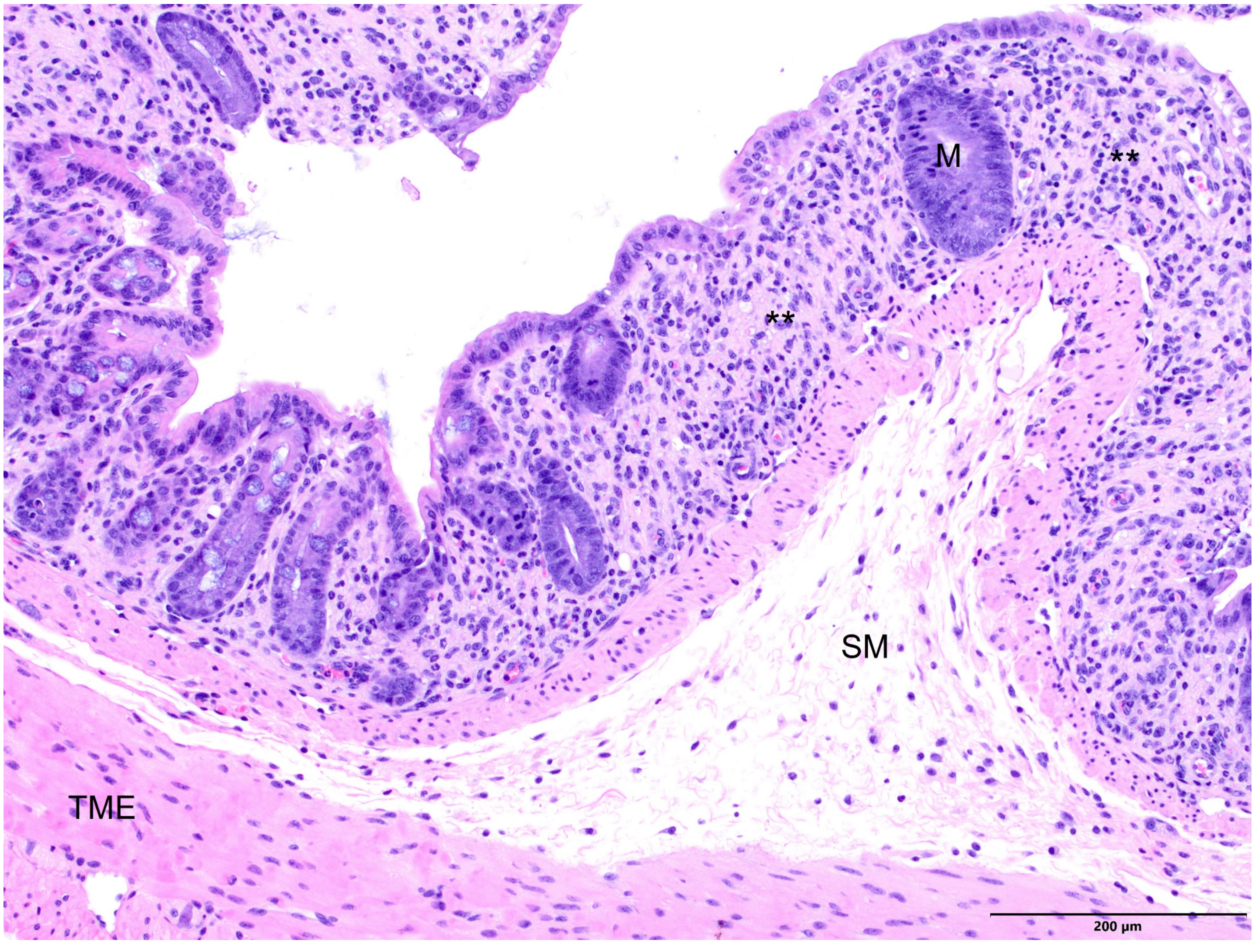

Supplement: Supplementary file 1 [file Data_Sheet_1.PDF]
